# Inclined Substrate Deposition of Nanostructured TiO_2_ Thin Films for DSSC Application

**DOI:** 10.3390/molecules26113122

**Published:** 2021-05-24

**Authors:** Lijian Meng, Tao Yang

**Affiliations:** 1 Centre of Innovation in Engineering and Industrial Technology, Instituto Superior de Engenharia do Porto, Instituto Politécnico do Porto, 4200-072 Porto, Portugal; 2 College of New Materials and New Energies, Shenzhen Technology University, Shenzhen 518118, China; yangtao@sztu.edu.cn

**Keywords:** dye-sensitized solar cells, DSSC, inclined substrate deposition, glancing angle deposition, TiO_2_ thin film, sputtering, nanostructure

## Abstract

Nanostructured TiO_2_ films were deposited onto Indium Tin Oxide (ITO) and glass substrates by dc reactive magnetron sputtering at different substrate inclination angles. The structural and optical properties of the deposited films were studied by X-ray diffraction, scanning electron microscopy and UV–Vis spectrophotometer, respectively. Dye-sensitized solar cells (DSSC) were assembled using these TiO_2_ films as photoelectrodes and the effect of the substrate inclination angle in the preparing process of TiO_2_ films on the DSSC conversion efficiency was studied.

## 1. Introduction

Titanium dioxide has been used in a wide range of applications because of its useful electrical and optical properties, such as high dielectric constant, high electrical resistivity, high refractive index, excellent optical transmittance in the visible range, non-toxicity, low cost and large band gap [1,2,3,4,5]. TiO_2_ film with a nanoporous structure is needed for applications in the solar cell and catalyst areas [6,7,8,9]. Oblique angle deposition, also called glancing angle deposition or inclined substrate deposition, generally produces film with a nanostructure. The oblique angle flux incidence enhances atomic shadowing and creates an inclined columnar microstructure under conditions of limited adatom diffusion. This technique is usually combined with the physical vapor deposition technique [10,11,12,13]. It is well known that the sputtering technique is a standard industrial production technique. The deposition parameters are very easy to control, and the deposited films generally have a good adhesion with the substrate. It is widely used for large area thin-film fabrications [14,15,16,17]. Generally, the glancing angle deposition technique is associated with evaporation processes. Recently, Gormier et al. demonstrated that a TiO_2_ nanostructure could be obtained with the sputtering technique by the substrate inclination [12]. However, they only got a nanostructure at 80° inclination with the post-annealing process. In this work, TiO_2_ films are prepared on the ITO and glass substrate combined with a tilted substrate. The effect of the substrate inclination angle on the structural and optical properties of deposited TiO_2_ films are reported. The dye-sensitized solar cells are assembled using these TiO_2_ films and the performance of the solar cells is also studied.

## 2. Experimental Details

TiO_2_ thin films were deposited on the commercial ITO (sheet resistance of 20 Ω per square) substrates by the dc reactive magnetron sputtering technique using a commercial sputtering system equipped with a turbo molecular pumping system. The target was a titanium metal disk (60 mm in diameter and 3 mm in thickness) with a purity of 99.99%. The chamber was pumping down to 1 × 10^−3^ Pa before the gases were introduced. The oxygen and argon gases (99.99% purities) were introduced into the chamber through the mass flow controllers. The oxygen partial pressure and the total sputtering pressure in the chamber were 0.5 Pa and 1.3 Pa, respectively. The sputtering was carried out using a constant current mode. The sputtering current was kept at 0.5 A and the sputtering power was about 190 W. In order to remove surface contaminants of the target, pre-sputtering was done for 20 min with a shutter covering the substrate. The substrate was fixed on a specially designed apparatus which can incline the substrate for different angles in relation to the horizontal. The distance between the target and the center of the substrate was kept at 60 mm. The substrate inclination angle was adjusted from 0° to 85°. However, the experimental results for the 30° and 45° inclination angles were very similar to that without inclination of the substrate. Therefore, they are not shown in the results. The deposition time was 180 min. The transmittance of the films was measured using a Jasco V-550 UV–Vis spectrophotometer (Tokyo, Japan). The XRD measurements were done using a Rigaku miniflex goniometer (30 kV, 15 mA) (Tokyo, Japan). The morphologies of the films were studied using a field emission scanning electron microscope (FE-SEM).

The deposited TiO_2_ films were sensitized with N719 (Ru(II)L2(NCS)2:2TBA, where L = 2,2′-bipyridyl-4,4′-dicarboxylic acid) dye by soaking the films in an ethanolic solution of N719 dye (0.5 mM) for 24 h at room temperature. The counter-electrode was made by sputtering Pt onto FTO glass and the electrolyte was composed of 0.1 M I_2_, 0.1 M LiI, 0.6 M 1-hexyl-3-methylimidazolium iodide, and 0.5 M 4-tert-butylpyridine in 3-methoxypropionitrile. The photocurrent-voltage measurements were carried out with a Princeton 2273 applied research electrochemical system (Oak Ridge, TN, USA), a 500 W Xenon lamp under AM 1.5 G illumination, and a water filter was used. The light intensity was adjusted to 100 mW/cm^2^. Cells with an active area of 0.15 cm^2^ were tested.

## 3. Results and Discussion

**Figure 1:**
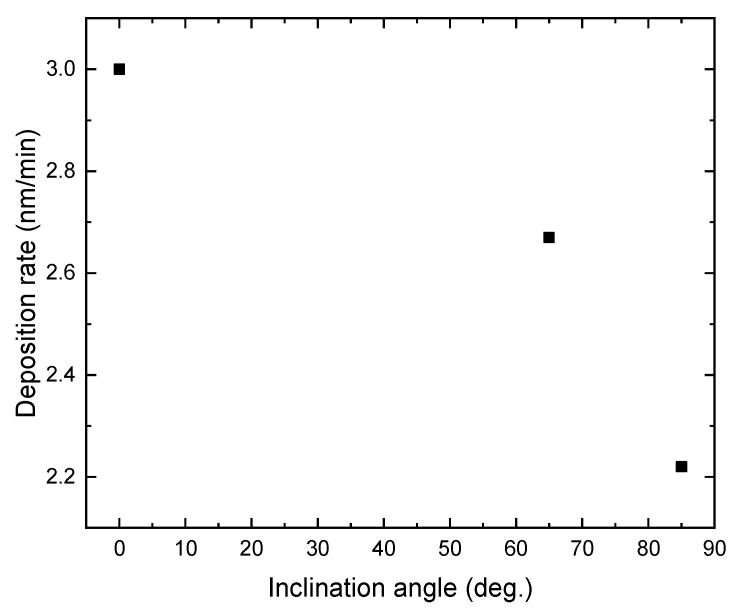
The deposition rate of TiO_2_ film as a function of the inclination angle of the substrate.

Figure 1 shows the deposition rate of TiO_2_ films prepared at different substrate inclination angles. The film thickness was measured by SEM and results are listed in Table 1. It can be seen that the deposition rate decreased as the inclination angle increased. Poxson et al. [18] have proposed an analytic model that accurately predicts the deposition rate of nanoporous films made by oblique-angle deposition. They predict a decrease of the deposition rate with increase of the substrate inclination angle, which has been proved by the experimental results of SiO_2_ and ITO films. The result shown in Figure 1 is in agreement with this prediction. Although the deposition rate for TiO_2_ films also decreased, the descent rate was lower than the theoretical expectation and experimental results for SiO_2_ and ITO films. This difference may result from the different deposition techniques. Generally, oblique-angle deposition is applied with the evaporation technique. In this work, the sputtering technique was used.

**Figure 2:**
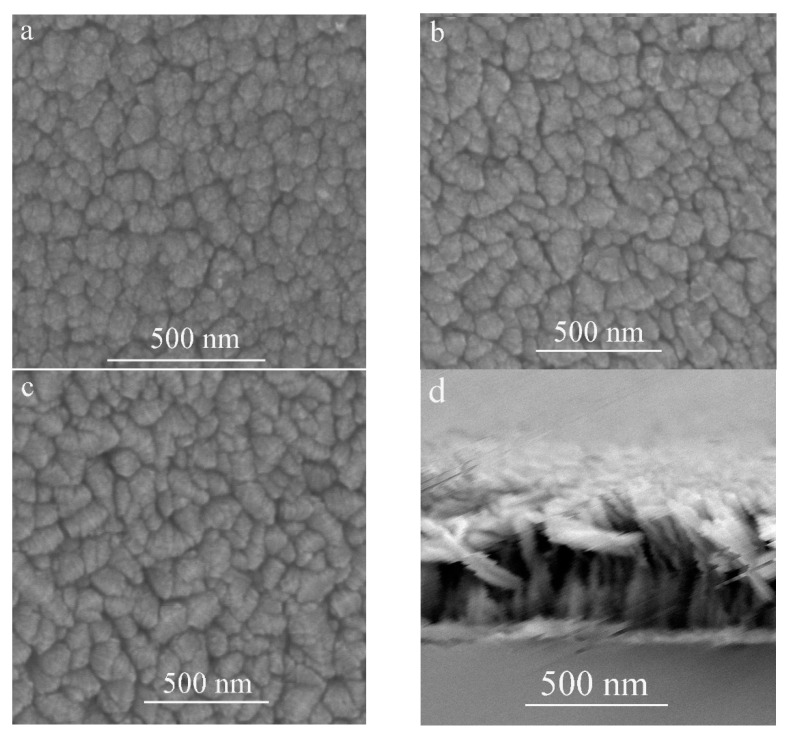
FE-SEM images of TiO_2_ films prepared at different substrate inclination angles ((**a**) 0°; (**b**) 65°; (**c**) 85°; (**d**) cross-section view of the sample prepared at 85° inclination angle).

The FE-SEM top-view images of the TiO_2_ film deposited at different substrate inclination angles are shown in Figure 2a–c. The cross-section view image of TiO_2_ film prepared at 85° substrate inclination angle is shown in Figure 2d. Our previous results have shown that a TiO_2_ nanorod structure can be formed at high sputtering pressure [17]. However, the inclination of the substrate angle was much more favorable for this structure, as can be seen from the top-view images. It can be seen that although the nanorod structure can be formed without substrate inclination, the inclination of the substrate was favorable for the separation of these nanorods as shown in Figure 2a–c, which means the porosity of the film increased with increase of the substrate inclination angle. A high substrate inclination angle will lead to more anisotropic atomic shadowing and result in more voids to be incorporated into the film [18].

**Figure 3:**
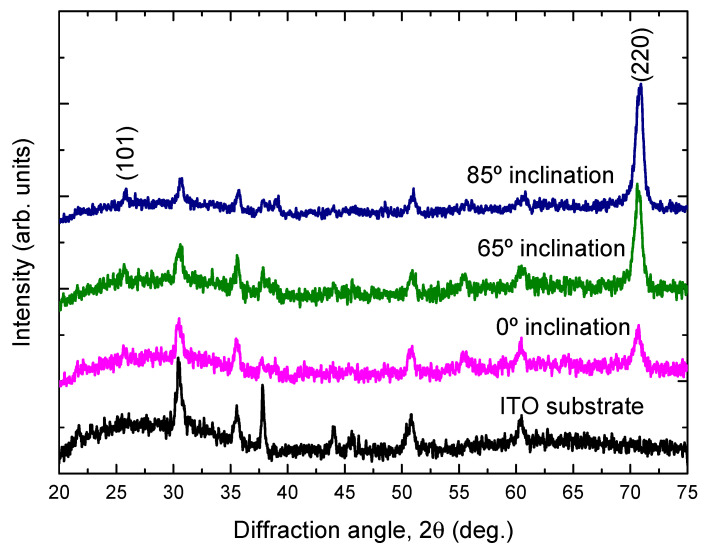
X-ray diffraction patterns of TiO_2_ films prepared at different substrate inclination angles. For comparison, the X-ray diffraction pattern of the ITO substrate is also shown in the figure.

**Figure 4:**
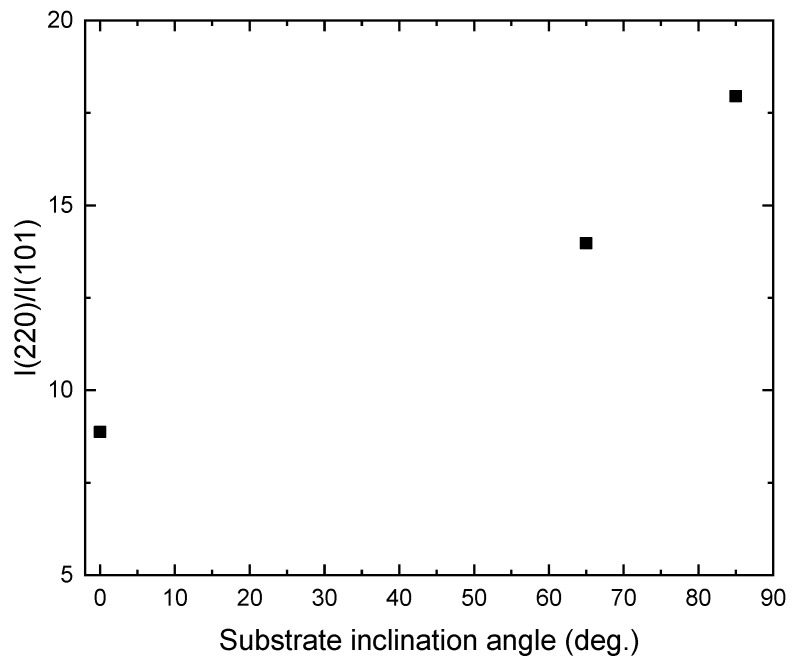
Variation of I(220)/I(101) with the substrate inclination angle.

Figure 3 shows the XRD patterns of the TiO_2_ films deposited onto ITO substrates at different substrate inclination angles. For comparison, the XRD pattern of the ITO substrate is also shown in the figure. Only the anatase phase of TiO_2_ is observed. All the films show a preferred orientation along the (220) direction. This preferred orientation is much enhanced with increase of the substrate inclination angle. The intensity ratio of (220) diffraction peak to (101) diffraction peak for TiO_2_ films prepared at different substrate inclination angles has been plotted in Figure 4. It can be seen that this ratio increased as the substrate inclination angle increased. It means that the substrate inclination is favorable for nanorod formation as the TiO_2_ nanorod grows along the (220) direction [19].

**Figure 5:**
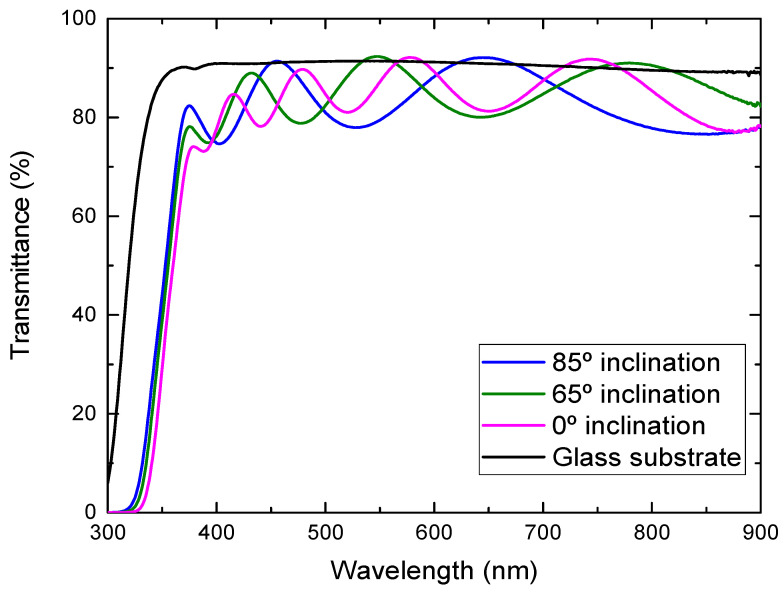
Optical transmittance of TiO_2_ films prepared at different substrate inclination angles.

**Figure 6:**
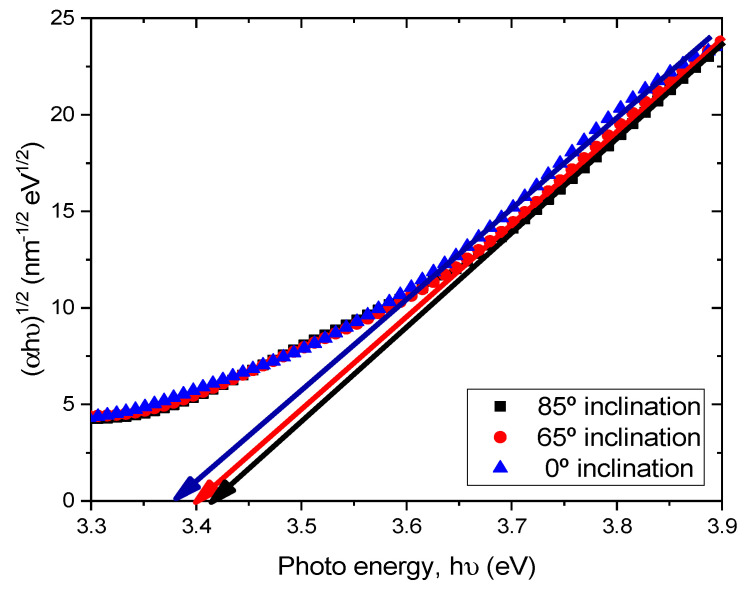
(αhν)^1/2^ as a function of the photon energy (hν) for TiO_2_ films prepared at different substrate inclination angles.

Figure 5 shows the optical transmission spectra of the TiO_2_ films deposited onto the glass substrates at different substrate inclination angles. The transmittance of the glass substrate without film is also shown in the figure. In some wavelength regions, the transmittance of the TiO_2_ films was higher than the bare glass substrate, which comes from the volume inhomogeneity of the deposited films. The inhomogeneity causes a small fluctuation of the refractive index and results in this phenomenon. It can be seen from the figure that the transmittance in the visible region was high for the film prepared at a high substrate inclination angle. The film prepared at a high substrate inclination angle was thinner than that prepared at a low inclination angle, which may cause a decrease of the transmittance. In addition, TiO_2_ film prepared at a high substrate inclination angle showed a better separated nanorod structure than that prepared at a low substrate inclination angle, which may also have contributed to the improvement of the optical transmission. It can be also seen from Figure 5 that the optical band gap moved to a short wavelength for the film prepared at a high substrate inclination angle. The optical band gap of TiO_2_ films can be estimated by the following equation:

(1) $$\alpha h\nu =A{\left(h\nu -E_{g}\right)}^{m}$$

where α is the absorption coefficient, hν is the photon energy, A is the constant which depends on the effective mass of the charge carrier in the material, Eg is the optical band gap and m is the exponent defined by the mechanism of the photon absorption of the semiconductor and is theoretically equal to ½ and 2 for direct and indirect allowed transition, respectively [20]. Therefore, by the extrapolation of the linear part of the curve (αhν)^2^ − hν or (αhν)^1/2^ − hν to the zero value of the ordinate, the optical band gap Eg can be obtained from the intercept of abscissa for direct and indirect transitions. As TiO_2_ is an indirect transition band gap material, (αhν)^1/2^ versus photon energy (hν) were plotted for TiO_2_ films prepared at different substrate inclination angles, as shown in Figure 6. It can be seen that the optical band gap was about 3.37 eV, 3.40 eV, and 3.42 eV for TiO_2_ films prepared at 0°, 65°, and 85° substrate inclination angles, respectively. As can be seen from the SEM images, the sample prepared at a high substrate inclination angle had a better nanostructure than that prepared at a low substrate inclination angle. This may have implications for the quantum confinement effect and result in a blue shift of the optical band gap.

**Figure 7:**
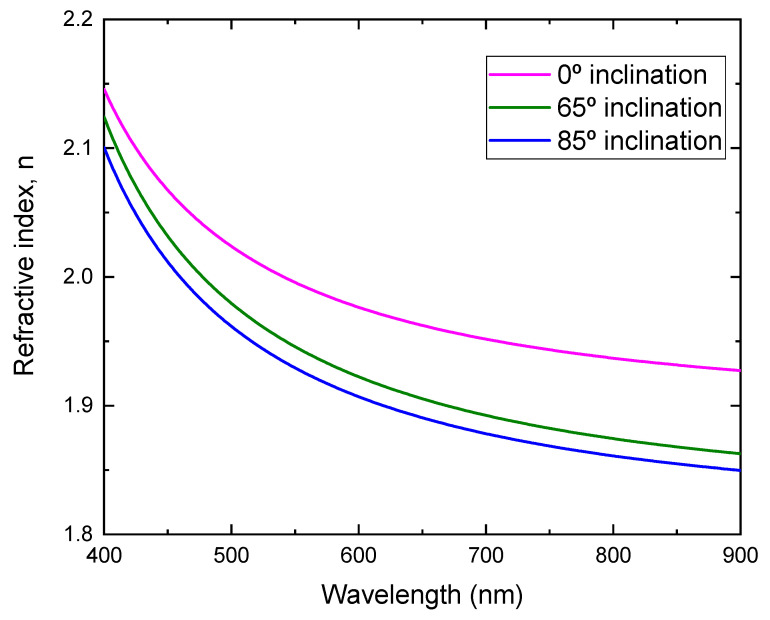
Refractive index of TiO_2_ films prepared at different substrate inclination angles.

By fitting the transmittance, the refractive index as a function of the wavelength for the films prepared at different substrate inclination angles is obtained, as shown in Figure 7. It can be seen that the film prepared at a low substrate inclination angle had a high refractive index. It is well known that the film packing density is related to the refractive index. High refractive index film has a high packing density. The high substrate inclination angle produces film with high porosity and results in a low refractive index. This structure was confirmed by SEM measurement, as shown in Figure 2.

**Figure 8:**
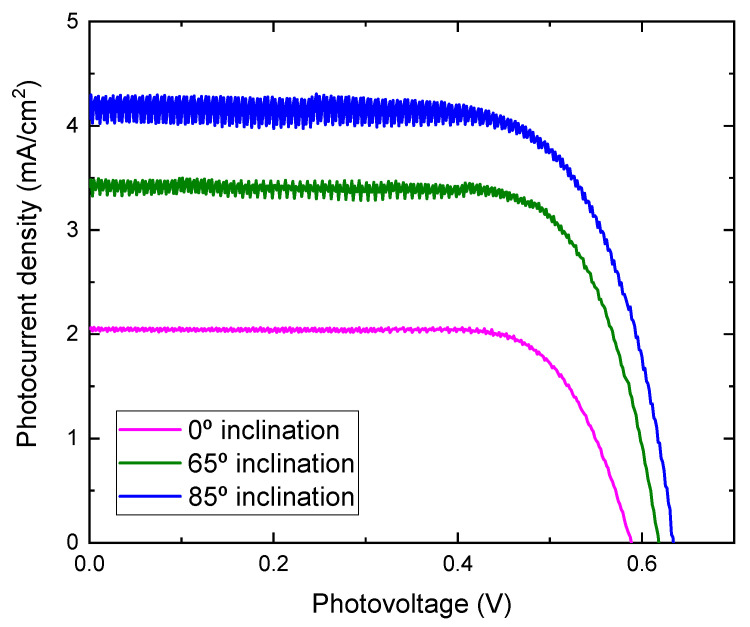
J-V curves for DSSC assembled with TiO_2_ films prepared at different substrate inclination angles.

**Figure 9:**
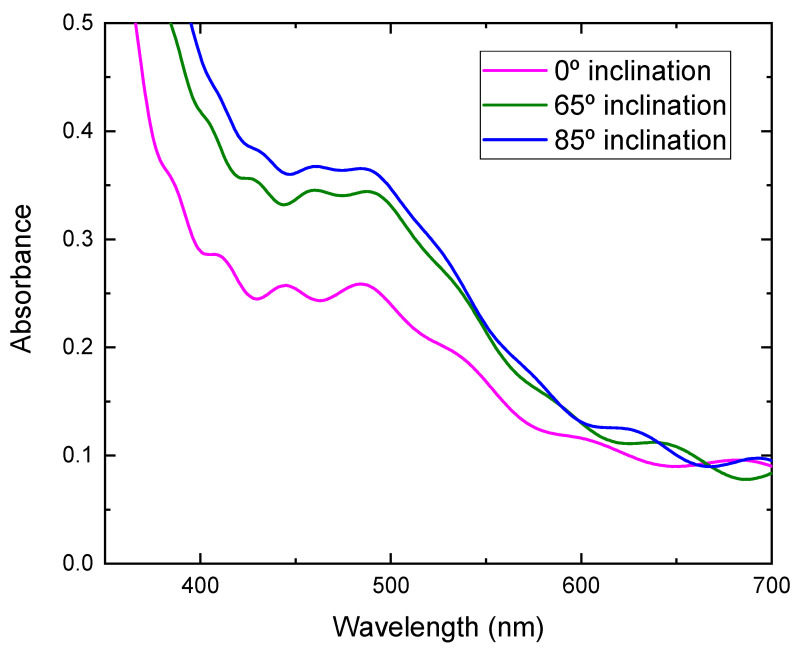
Absorbance of TiO_2_ films prepared at different substrate inclination angles after dye absorption.

Photocurrent density-voltage characteristics of DSSCs assembled with TiO_2_ films prepared at different substrate inclination angles as photoelectrodes are shown in Figure 8. The noise increased as the inclination angle increased. As the inclination angle increased, the deposition rate decreased (Figure 1). Low deposition rate results in a decrease of the thickness and may cause the increase of noise. The photoelectric conversion efficiency was calculated using the equation:

(2) $$\eta =\frac{J_{\mathrm{sc}}V_{\mathrm{oc}}\mathrm{FF}}{P_{\mathrm{in}}}\times 100$$

where η is the conversion efficiency, J_sc_ is short-circuit current density, which depends on the charge injection and transportation, V_oc_ is the open circuit voltage, which is most likely related to the difference between the Fermi level of the semiconductor electrode and redox potential in the electrolyte, and FF is the fill factor, which is related to functioning of the TiO_2_/electrolyte interface. The higher the recombination of conduction band electrons with the electrolyte, the lower will be FF [21], and P_in_ is incident light energy. The values of V_oc_, J_sc_, FF, and calculated conversion efficiency are listed in Table 1. It can be seen from Figure 8 that the photocurrent density improved greatly by using TiO_2_ film prepared at a high substrate inclination angle. Although the open circuit voltage and the fill factor also showed a small variation for DSSC assembled with TiO_2_ film prepared at different substrate inclination angles, the conversion efficiency was dominated by photocurrent density, as shown in Table 1. The photocurrent density is related to both electron density and electron mobility. By increasing the substrate inclination angle, the porosity of the deposited TiO_2_ film increased, which was confirmed both by SEM measurement and by the calculation of the refractive index. The increase of the porosity of the TiO_2_ film improves the absorption of the dye molecules and results in the increase of the electron density. The absorbance of TiO_2_ films prepared at different substrate inclination angles, after they are sensitized by dye, were measured as shown in Figure 9. It clearly shows that the TiO_2_ film prepared at a high substrate inclination angle had high absorbance after dye absorption. This means that the TiO_2_ prepared at a high substrate inclination angle can absorb more dye molecules than that prepared at a low substrate inclination angle, which will produce a high electron density and result in a high photocurrent density. Further, the nanorod crystallinity improved as the substrate inclination angle increased, which was shown in XRD patterns. This improvement of the crystallinity may result in an increase of the electron mobility and then an increase of the photocurrent density too. It can be seen from Table 1 that the conversion efficiency increased significantly by using TiO_2_ film prepared at an 85° substrate inclination angle as a photoelectrode, which means it is an efficient way to improve the conversion efficiency of dye-sensitized solar cells.

## 4. Conclusions

Nanostructured TiO_2_ films were deposited onto ITO and glass substrates by dc reactive magnetron sputtering at different substrate inclination angles. The deposition rate decreased with an increasing substrate inclination angle. All the films showed an anatase phase with a preferred orientation along the (220) direction and a nanorod structure. As the substrate inclination angle increased, the preferred orientation along the (220) direction was enhanced and the voids between nanorods increased; the optical band gap increased and the refractive index decreased. Dye-sensitized solar cells were assembled with these TiO_2_ films. Conversation efficiency was dominated by photocurrent density. By using TiO_2_ film prepared at an 85° substrate inclination angle as an electrode, the conversion efficiency was double compared to that using TiO_2_ film prepared without substrate inclination as an electrode.

## Figures and Tables

**Table 1:** Photovoltaic performance of the dye-sensitized solar cells (DSSC) based on TiO_2_ electrodes prepared at different inclination angles.

Inclination Angle (°)	V_oc_ (V)	J_sc_ (mA/cm^2^)	FF	η (%)	Thickness (nm)
0	0.59	2.06	0.75	0.91	540
65	0.62	3.48	0.73	1.58	480
85	0.63	4.29	0.70	1.89	400

## Data Availability

Not applicable.

## Sample Availability

Samples of the compounds are not available from the authors.

## References

[1] Guillén C., Montero J., Herrero J. (2014). Anatase and rutile TiO_2_ thin films prepared by reactive DC sputtering at high deposition rates on glass and flexible polyimide substrates. J. Mater. Sci..

[2] Jiang W., Cui H., Song Y. (2018). Electrochemical corrosion behaviors of titanium covered by various TiO_2_ nanotube films in artificial saliva. J. Mater. Sci..

[3] Zeinali M., Jaleh B., Vaziri M.R.R., Omidvar A. (2019). Study of nonlinear optical properties of TiO_2_—polystyrene nanocomposite films. Quantum Electron..

[4] Wang X., Lai M., Gao R., Huang X., Zhao Z., Yang Y., Zheng G., Ma Y. (2019). Ultra-smooth TiO_2_ thin film based optical humidity sensor with a fast response and recovery. Appl. Opt..

[5] Hassanien A., Akl A.A. (2020). Optical characterizations and refractive index dispersion parameters of annealed TiO_2_ thin films synthesized by RF-sputtering technique at different flow rates of the reactive oxygen gas. Phys. B Condens. Matter.

[6] Jiang Y., Chen W.F., Koshy P., Sorrell C.C. (2019). Enhanced photocatalytic performance of nanostructured TiO_2_ thin films through combined effects of polymer conjuga-tion and Mo-doping. J. Mater. Sci..

[7] Tuckute S., Varnagiris S., Urbonavicius M., Lelis M., Sakalauskaitea S. (2019). Tailoring of TiO_2_ film crystal texture for higher photocatalysis efficiency. Appl. Surf. Sci..

[8] Sampaio D.M., Babu R.S., Costa H.R.M., De Barros A.L.F. (2018). Investigation of nanostructured TiO2 thin film coatings for DSSCs application using natural dye extracted from jabuticaba fruit as photosensitizers. Ionics.

[9] Umale S., Sudhakar V., Sontakke S.M., Krishnamoorthy K., Pandit A.B. (2019). Improved efficiency of DSSC using combustion synthesized TiO_2_. Mater. Res. Bull..

[10] Hu Z., García-Martín J.M., Li Y., Billot L., Sun B., Fresno F., García-Martín A., González M.U., Aigouy L., Chen Z. (2020). TiO_2_ Nanocolumn Arrays for More Efficient and Stable Perovskite Solar Cells. ACS Appl. Mater. Interfaces.

[11] Rodrigues M.S., Borges J., Proença M., Pedrosa P., Martin N., Romanyuk K., Kholkin A.L., Vaz F. (2019). Nanoplasmonic response of porous Au-TiO_2_ thin films prepared by oblique angle deposition. Nanotechnology.

[12] Cormier P.-A., Dervaux J., Szuwarski N., Pellegrin Y., Odobel F., Gautron E., Boujtita M., Snyders R., Boujita M. (2018). Single Crystalline-like and Nanostructured TiO_2_ Photoanodes for Dye Sensitized Solar Cells Synthesized by Reactive Magnetron Sputtering at Glancing Angle. J. Phys. Chem. C.

[13] Wang B., Qi H., Liu Z., Jin Y., Wang H., Yuan J., Zhao J., Shao J. (2017). Structure, chemical state and photocatalytic activity of TiO_2−x_ nanostructured thin films by glancing angle deposition technique. J. Alloys Compd..

[14] Jeong J.-A., Kim H.-K. (2011). Thickness effect of RF sputtered TiO_2_ passivating layer on the performance of dye-sensitized solar cells. Sol. Energy Mater. Sol. Cells.

[15] Kang S.H., Kang M.-S., Kim H.-S., Kim J.-Y., Chung Y.-H., Smyrl W.H., Sung Y.-E. (2008). Columnar rutile TiO_2_ based dye-sensitized solar cells by radio-frequency magnetron sputtering. J. Power Sources.

[16] Lee C.H., Kim K.H., Choi H.W. (2012). Enhancing Efficiency of Dye-Sensitized Solar Cells Using TiO_2_ Composite Films and RF-Sputtered Passivating Layer. Mol. Cryst. Liq. Cryst..

[17] Meng L., Ma A., Ying P., Feng Z., Li C. (2011). Sputtered highly ordered TiO_2_ nanorod arrays and their applications as the electrode in dye-sensitized solar cells. J. Nanosci. Nanotechnol..

[18] Poxson D.J., Mont F.W., Schubert M.F., Kim J.K., Schubert E.F. (2008). Quantification of porosity and deposition rate of nanoporous films grown by oblique-angle deposition. Appl. Phys. Lett..

[19] Meng L., Chen H., Li C., Dos Santos M.P. (2015). Preparation and characterization of dye-sensitized TiO_2_ nanorod solar cells. Thin Solid Films.

[20] Tauc J., Grigorovici R., Vancu A. (1966). Optical Properties and Electronic Structure of Amorphous Germanium. Phys. Status Solidi.

[21] Thavasi V., Renugopalakrishnan V., Jose R., Ramakrishna S. (2009). Controlled electron injection and transport at materials interfaces in dye sensitized solar cells. Mater. Sci. Eng. R Rep..

